# Genetic Affinities within a Large Global Collection of Pathogenic *Leptospira*: Implications for Strain Identification and Molecular Epidemiology

**DOI:** 10.1371/journal.pone.0012637

**Published:** 2010-08-27

**Authors:** Kishore Nalam, Ahmed Ahmed, Sundru Manjulata Devi, Paolo Francalacci, Mumtaz Baig, Leonardo A. Sechi, Rudy A. Hartskeerl, Niyaz Ahmed

**Affiliations:** 1 Pathogen Biology Laboratory, Department of Biotechnology, School of Life Sciences, University of Hyderabad, Hyderabad, India; 2 WHO/FAO/OIE and National Collaborating Centre for Reference and Research on Leptospirosis, Department of Biomedical Research, Royal Tropical Institute (KIT), Amsterdam, The Netherlands; 3 Central Food Technological Research Institute, Mysore, India; 4 Department of Zoology and Evolutionary Genetics, University of Sassari, Sassari, Italy; 5 Government Vidarbha Institute of Science and Humanities, Amravati, Maharashtra, India; 6 Department of Biomedical Sciences, University of Sassari, Sassari, Italy; Cairo University, Egypt

## Abstract

Leptospirosis is an important zoonosis with widespread human health implications. The non-availability of accurate identification methods for the individualization of different *Leptospira* for outbreak investigations poses bountiful problems in the disease control arena. We harnessed fluorescent amplified fragment length polymorphism analysis (FAFLP) for *Leptospira* and investigated its utility in establishing genetic relationships among 271 isolates in the context of species level assignments of our global collection of isolates and strains obtained from a diverse array of hosts. In addition, this method was compared to an in-house multilocus sequence typing (MLST) method based on polymorphisms in three housekeeping genes, the *rrs* locus and two envelope proteins. Phylogenetic relationships were deduced based on bifurcating Neighbor-joining trees as well as median joining network analyses integrating both the FAFLP data and MLST based haplotypes. The phylogenetic relationships were also reproduced through Bayesian analysis of the multilocus sequence polymorphisms. We found FAFLP to be an important method for outbreak investigation and for clustering of isolates based on their geographical descent rather than by genome species types. The FAFLP method was, however, not able to convey much taxonomical utility sufficient to replace the highly tedious serotyping procedures in vogue. MLST, on the other hand, was found to be highly robust and efficient in identifying ancestral relationships and segregating the outbreak associated strains or otherwise according to their genome species status and, therefore, could unambiguously be applied for investigating phylogenetics of *Leptospira* in the context of taxonomy as well as gene flow. For instance, MLST was more efficient, as compared to FAFLP method, in clustering strains from the Andaman island of India, with their counterparts from mainland India and Sri Lanka, implying that such strains share genetic relationships and that leptospiral strains might be frequently circulating between the islands and the mainland.

## Introduction

Leptospirosis caused by the pathogenic spirochetes of the genus *Leptospira* is the most widespread zoonosis in the world [1]–[5] where the number of severe cases probably exceeds 500,000 per year. Case-fatality rates are >10% and >50% in patients who develop acute hepato-renal failure or pulmonary hemorrhage syndrome, respectively. Pathogenic *Leptospira* consist of about 300 distinct antigenic types referred to as serovars, which vary with their carrier animal species [5]–[7]. *Leptospira* are maintained in the genital tract and renal tubules of wild and domestic animals and are excreted with urine into the environment where they can survive for several months depending on favorable conditions such as warm, humid environment with a neutral to slightly alkaline pH [2], [5], [7]. Infection of accidental hosts occurs by direct contact with the infected animals or their urine or indirectly via urine-contaminated environment. Accidental hosts develop clinical manifestations with a varying degree of severity and potentially leading to death [4]. *Leptospira* is a genus within the order Spirochaetales, an early branch in eubacterial evolution that, as a group, has unusual patterns of genetic organization. Analyses based on DNA composition have identified 20 *Leptospira* species with seven pathogenic species, which are *L. interrogans*, *L. borgpetersenii, L. santarosai*, *L. noguchi*, *L. weilii*, *L. kirschneri* and *L. alexanderi* comprising the main agents of leptospirosis [1], [2], [6]. The pathogenic *Leptospira* spp. form a common branch in evolution, distinct from saprophytic *Leptospira* [8]. Recent reports identified an increasing intermediate group of *Leptospira* isolated from animals and humans with no or mild clinical symptoms [8]–[12]. The significance of this intermediate group in leptospirosis is yet unknown.

Genome sequencing has revealed a high-level plasticity of *Leptospira* genomes [13], [14]. It has been proposed that *Leptospira* had a common progenitor with a genome resembling to that of *L. biflexa*. Mammalian infection potential could be associated with the acquisition of genes [15] expanding *Leptospira*'s capacity to survive host-determined environmental conditions while subsequent genome reduction increased host dependence.

Considering its unusual high antigenic and genetic flexibility, the genus *Leptospira* presents an extremely important research model for the understanding of pathogen evolution. However, focused *Leptospira* evolution research is scarce up to date.

The enormous repertoire of *Leptospira* serovars is mainly based on ever-changing surface antigens, notably the LPS. This presents an unreliable scenario of strain diversity and makes the serological approach difficult to track strains whose molecular identity keeps changing according to the host and environmental niches they inhabit and cross through. Multilocus sequence typing (MLST) [16], [17], fluorescent amplified fragment length polymorphism (FAFLP) [18] and multilocus variable number of tandem repeats analysis (MLVA) [19] are the first genome sequence based molecular approaches having already established promise in unraveling *Leptospira* phylogeny, albeit in studies on limited strain panels or strains with restricted geographic prevalence. These methods have their advantages and disadvantages: MLST makes use of sequences deduced from PCR amplified DNA segments and thus depends on the success of amplification, which in turn depends on the annealing efficiency of the PCR primers. Sequence drift between *Leptospira* species will thus limit the applicability of MLST, particularly to the strains that fall in genetically distant branches. Amplification in FAFLP does not depend on the bacterial sequence composition and thus has a wide applicability. The drawback is that FAFLP requires high quality reagents and purified, concentrated genomic DNA. MLVA methods generally do not expand beyond *L. interrogans* or have limited flexibility to extend to all pathogenic and non-pathogenic species [20]. Given these issues, it would be relevant to test these methods in conjunction on a defined, global collection of strains and to see how they complement and supplement each other.

In the present study, we describe the genetic affinities and ancestral origins among the members of a strong 271 strains collection representing global dispersal and corresponding to a diverse array of hosts (Table S2). We applied both MLST and FAFLP with a focus on all pathogenic species. We further dissected diversity and composition of *L. interrogans* (being the largest subgroup within our collection), by a fluorescent MLVA technique. In addition, we studied genetic linkages among strains obtained from geographically close regions; such as the gene flow among *L. interrogans* within the Indian sub-continent.

## Materials and Methods

### Bacterial strains and genomic DNA samples

We included 271 *Leptospira* strains and isolates in the phylogenetic study. The strains and their sources are listed in supplementary information, tables S1 and S2. All the strains were cultured by the WHO reference laboratory at the KIT Biomedical Research Centre at The Royal Tropical Institute, Amsterdam, The Netherlands and at the Veterinary Sciences Division (VSD), The Queen's University of Belfast, United Kingdom and the WHO reference centre at Port Blair, India. The bacterial isolates were obtained over the last few years as a part of routine diagnostic/epidemiological investigations and they do not correspond to any cohorts or recruited patients/individuals (supplementary information, tables S1 and S2). Hence, they did not require any consents or ethics approval. Even then, the Institutional Biosafety Committee of the University of Hyderabad approved the study protocols. The study also has approvals from the Institutional Review Boards of all the participating institutions. *Leptospira* were grown to late log phase, harvested by centrifugation and genomic DNA was extracted using a QIAamp DNA mini kit (Qiagen, Germany) following the manufacturer's instructions.

### Gene loci, nucleotide sequences and data access

The rational for the choice of the 6 candidate gene loci, their co-ordinates and amplification conditions etc. as needed for the design, testing and validation of the MLST scheme have all been detailed previously by us [8], [16]. The *secY* gene and its resolution power in comparison with other signature loci such as *rrs* have been already determined in a previous study from our extended group [8]. The relevant sequence records are available via GenBank accession numbers EU365895-EU365966 and EU357938–EU358070. For other gene loci, prototype sequences needed for the design of MLST were obtained from the genomes of *L. interrogans* serovar Lai (NC_004342 and NC_004343) and *L. interrogans* serovar Copenhageni (NC_005823 and NC_005824) The multi locus sequences of all the 271 isolates obtained as a part of this study are available in full under supporting information (Table S2).

### FAFLP method and phylogenetic analysis

Whole genome fingerprinting based on FAFLP genotyping was performed as described previously [18]. Briefly, the profiling of whole genome micro-restriction fingerprints with *Eco*RI/*Mse*I enzymes using fluorescence tagged primer pairs *Eco*RI+A/*Mse*I+0 and *Eco*RI+G/*Mse*I+0 was performed for all the strains. The PCR amplified fragments for each of the strains were then subjected to electrophoretic separation on a 5% acrylamide gel on an ABI Prism automated DNA sequencer and scoring of the fluorescent markers was done using the same DNA analysis workstation (ABI Prism 3100 DNA sequencer). Cluster analysis of DNA profiles was conducted on the basis of fingerprint characteristics scored in the form of a binary table for the presence and absence of alleles within the bins generated for fragment sizing [18]. Phylogenetic tools within MEGA 3.0 were used to generate Neighbor-joining trees with bootstrapping as described earlier [21], [22].

### MLST method and phylogenetic analysis

Six 600 bp-long regions from six genes spread throughout the genome were amplified by PCR and sequenced exactly as described previously [3]. Sequencing was performed on the two strands, using the DNA sequencer (see above). PCR and direct sequencing were performed at least twice to determine and confirm the DNA sequences for each isolate. Consensus sequence for each of the samples was generated using Genedoc (version 2.6.002). Multiple alignments of sequenced nucleotides were carried out using Clustal X (version 1.81). Bifurcating Neighbor-joining trees were constructed in MEGA 3.0 using bootstrapping at 10000 bootstrap trials and through Kimura-2 parameter [21], [22].

### Network analysis based on FAFLP data

Network analysis using the program Network 4.5.0.0 (http://www.fluxus-engineering.com) was performed on MLST sequences and on FAFLP data. In particular, the median-joining algorithm, which can handle large data sets and multistate characters, was used [23]. Because of a program limitation, that it cannot handle more than 1000 polymorphic sites at once, we performed the analysis separately on two exact halves of the concatenated product (comprising of the multilocus sequences). This partition was neither necessary for FAFLP data nor for the *L. interrogans* subsample, which presented reduced numbers of polymorphisms; consequently, these datasets were analyzed in a complete form.

### Phylogenetic reconstruction by Bayesian Morkov Chain Monte Carlo (MCMC) approach using MLST data

The MLST data were subjected to Bayesian MCMC analysis using BEAST version 1.5.2 [24]. The most important feature of Bayesian MCMC analysis using BEAST is that it offers rooted phylogeny. While constructing the phylogeny we used relaxed molecular clock approach [25]. Both coalescent constant population size and Yule speciation tree prior were employed. Two independent runs for each model were achieved for 30000000 steps and sampled every 1000 steps. The first 100000 steps of each run were discarded as burn-in. The tree was annotated using the program TreeAnnotator v1.5.2 (http://tree.bio.ed.ac.uk/). Finally, the annotated tree thus obtained was viewed and saved using the program FigTree v1.2.2 (http://tree.bio.ed.ac.uk/).

### Automated MLVA analysis of the *L. interrogans* subsample

Multilocus variable number of tandem repeats analysis (MLVA) was carried out essentially as described previously by Majed *et al*. [19] except that the method was adopted for automated sequencer(s) by incorporating fluorescent labels into the reverse primers corresponding to all the loci previously tested [19]. Samples were analyzed on an automated DNA sequencer (ABI Prism 3100) and allele calling/binning was performed in a binary format as described previously for the FAFLP analysis [18]. MLVA data were used in MEGA 3.0 to generate phylogenetic trees.

## Results and Discussion

### FAFLP and MLVA as applied to the leptospiral genomes

**Figure 1:**
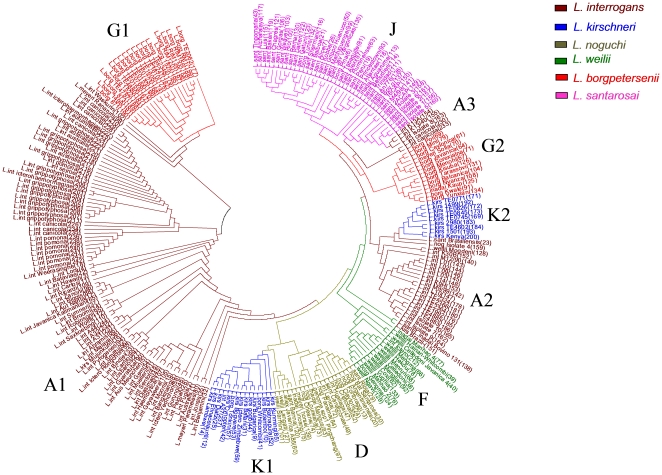
Genetic relatedness among *Leptospira* isolates based on FAFLP analysis. (See supplementary information, tables S1 and S2 for details.) Clades roughly corresponding to different species types have been marked in different colors. Major clades and minor clades have been identified by code names (A to K) which overlap with the designation of similar clades identified by MLST technique. Identities of individual isolates need not be comprehensible in the tree itself, but they can be read clearly in the supplementary Table S2. The phylogenetic tree was rendered and visualized by MEGA3.0 software.

**Figure 2:**
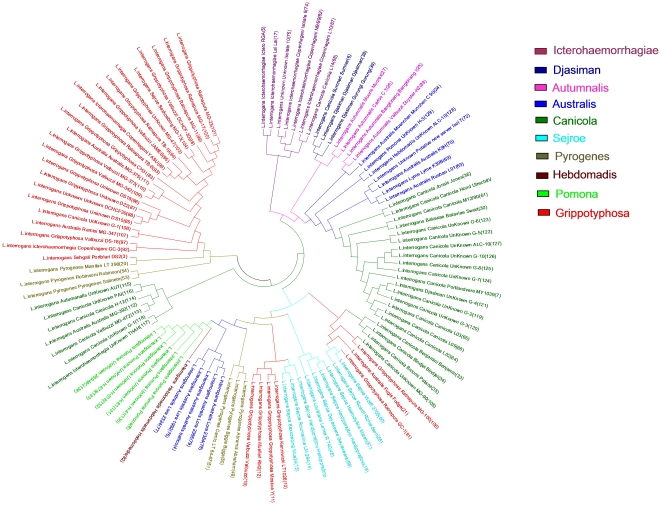
Genetic analysis of *L. interrogans* subsample based on MLVA analysis. Clusters corresponding to individual serogroups have been identified by different colors which have no correspondence with the color code of clusters shown in Figures 1 and 3. Please refer to supplementary information (Table S2) for details of the *L. interrogans* samples analyzed here. The phylogenetic tree was generated and visualized by using MEGA3.0 software.

Whole genome micro restriction patterns as scanned by the FAFLP method have revealed quite complex and confusing genetic affinities among various *Leptospira* species (Figure 1). This was not surprising given the resolution power of the FAFLP technique [18]. Nevertheless, plausible ancestral associations were found in terms of co-clustering of *L. kirschneri* and *L. interrogans*; *L. borgpetersenii* with *L. santarosai* and *L. noguchi* with *L. kirschneri*. Further analysis of the bifurcating Neighbor-joining tree revealed broadly species-specific clusters although clade-switching by a few strains in almost each of the clusters was clearly evident with respect to their projected genomic DNA-based species status. Thus, more than one cluster were observed for *L. interrogans, L. kirschneri,* and *L. borgpetersenii*. Further, this splitting of the clusters was not in agreement with geographical descent or the host species types, although we believe that discreet genetic associations arising due to recombinational events could lead to such subclustering. We tested this phenomenon by sub classifying the *L. interrogans* group by MLVA analysis. The tree based on MLVA (Figure 2) revealed embranchment broadly conforming to the serotypical positions and corresponding to the serogroups Icterohaemorrhagiae, Djasiman, Autumnalis, Australis, Canicola, Sejroe, Pyrogenes, Hebdomadis, Pomona, and Grippotyphosa. Containing this great diversity of serogroups within just 2 clusters should be seen as an appreciable specificity of the FAFLP method, which could be tapped for the investigation of small, regional outbreaks as previously shown by some of us [18]. However, in our opinion, when we seek replacement of serotyping as a tool for individualization, this sensitivity is not sufficient and perhaps FAFLP is not capable of distinguishing between the outcomes of genetic recombination or erroneous serotyping. Employing therefore a method that uses housekeeping genes, such as MLST appears to be inevitable.

### MLST analysis as applied to the *Leptospira* gene pool

**Figure 3:**
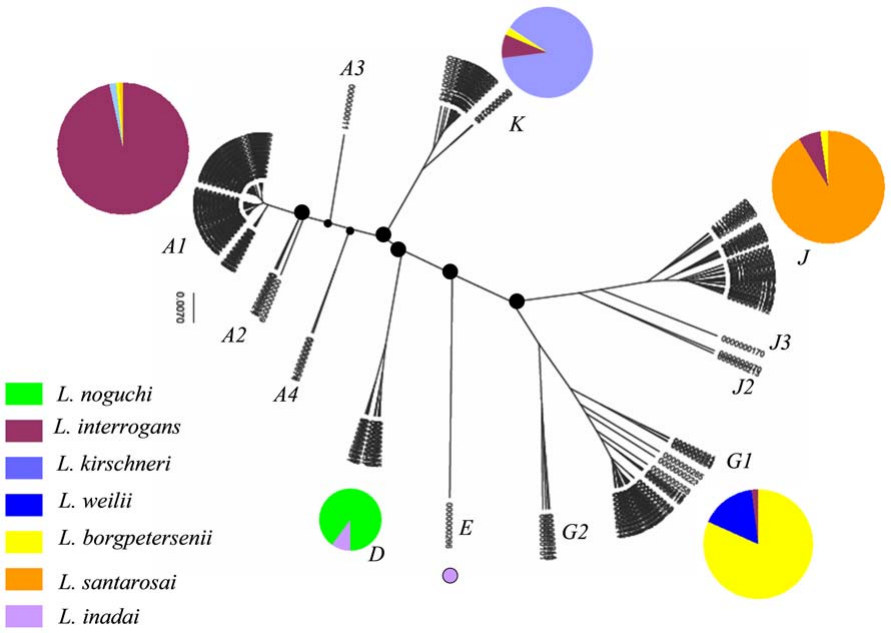
Genetic clustering and Bayesian inferred phylogeny of *Leptospira* isolates and strains based on MLST analysis. (See supplementary information, tables S1 and S2 for detailed information on all the isolates/strains analyzed.) Clades or embranchment corresponding to different species types have been marked in different colors. Major clades and minor clades have been identified by code names (A to K) which overlap with the designation of similar clades identified by FAFLP technique. Identities of individual isolates need not be comprehensible in the tree itself, but they can be read clearly in the supplementary Table S2. The analysis was performed using BEAST version 1.5.2, the tree was annotated using TreeAnnotator v1.5.2 and was visualized through FigTree v1.2.2.

MLST analysis of all the 271 isolates revealed a highly organized phylogenetic tree (Figure 3) with no split clusters or widespread clade switching as seen with FAFLP. Analysis with MEGA 3.0 (N-J) or Median Joining by Network did not change the tree topology or the composition of various branches. However, in order to reproduce and validate the associations in another robust manner and to know the extent of genetic affinities among various branches we performed Bayesian MCMC analysis (see the Materials and Methods section).

Both coalescent constant population size and Yule speciation tree prior yielded similar topology. The Bayesian inference (BI) tree (Figure 3) reveals that the global pathogenic *Leptospira* in current study split into twelve embranchment. These twelve embranchments show five distinct major clades, namely, A or A1, D, G or G1, J or J1, K and their minor (sub) clades designated as A2, A3, A4, G2, J2 and J3. All these major and minor clades were well supported by 95% highest posterior density (HPD) intervals. Of note, the BI tree was rooted with *L. inadai,* whose intermediate nature in the *Leptospira* systematics is well supported by earlier studies [8], [26]. In the BI tree constructed from 271 global isolates, the *L. interrogans* designated as clade A in Figure 3 comprises the largest collection of isolates and emerged as a tight cluster comprising of 123 isolates. Further, four visible sub-branching within the *L. interrogans* clade could perhaps suggest strain specific or host/environment specific genetic alterations. The other major clades found were *L. santarosai* and *L. kirschneri* designated as J and K respectively. Interestingly, the clade containing *L. borgpetersenii* isolates split into one major clade designated as G1 and a minor clade, G2. Both these major and minor clades show clustering with *L. weilii* isolates. The clade D with sole inclusion of *L. inadai* comprises entirely of *L. noguchi* strains. The minor clades A2 and A4 include four and three *L. interrogans* isolates respectively and behave as outliers. Taking into account the genetic distance, the minor clade A4 shows affinities towards *L. kirschneri* and not towards *L. interrogans.* In terms of genetic distance, the position of a sole *L. interrogans* isolate depicted as branch A3 in the tree lies intermediate between the *L. interrogans* and *L.kirschneri*. Thus the minor clade A2 which harbors two isolates from rodents (one each from a bandicoot and a necked field mouse) seems to be the most recent plausible ancestor of the present day *L. interrogans* found in varied hosts. The role of rodents as the reservoir of *Leptospira* needs no explanation. Further, *L. noguchi* shows closer genetic affinity towards *L. kirschneri* than *L. interrogans.* The basal position of *L. noguchi* cluster in the vicinity of the *L. kirschneri* and *L. interrogans* clusters is suggestive of the ancestral nature of *L. noguchi* or in a simpler way, *L. kirschneri* and *L.interrogans* may have both originated from *L. noguchi* or *L. noguchi* like ancestor. In contrast to the less distinct evolution of *L. kirschneri* and *L. interrogans*, the evolution of *L. santarosai, L. borgpetersenii and L. weilii* is marked by more distinct speciation events along the intermediate *L. inadai* or *L. inadai* like ancestor. The minor clade G2, comprises of two *L. weilii* and one *L. borgpetersenii* and *L.interrogans* isolate each. From the basal position of *L. weilii* in the minor clade G2 and in the major clade G1, it seems that *L. weilii* is the most recent common ancestor of the *L. santarosai* and *L. borgpetersenii* or *L. santarosai* and *L.borgpetersenii* originated from *L. weilii* or *L. weilii* like ancestor.

Apart from systematics, the inferred tree also reveals instances of unusual clustering. One of the isolates serologically typed as *L. interrogans* clustered with the *L. kirschneri* clade. Similarly, three *L. interrogans* serovars clustered with the *L. santarosai* clade. The tree also shows tight clustering of single *L. borgpetersenii and L. meyeri* isolates with the *L. interrogans* cluster. Also, two serologically classified *L. kirschneri* isolates clustered with the *L. interrogans* clade. Taking into consideration the genetic distances between these five major clades, clade switching seems to be feasible between *L. interrogans* and *L. kirschneri* and not between *L. interrogans, L. santarosai* and *L. borgpetersenii.* Such unusual clustering(s) could only be explained by either incorrect serological typing or clade switching due to horizontal gene transfer (HGT). A further mechanistic dissection of these isolates based on whole genome sequence analysis could shed some light on their genome evolution trends and the extent of HGT occurring within the *rfb* cluster that encodes much of the surface antigens responsible for determination of their serotype.

In summation of our analyses above (supplementary information, tables S1 and S2), it is possible to espouse that the *L. interrogans*, *L. kirschneri* and probably *L. borgpetersenii* are ubiquitous species. Within this, *L. interrogans* seems to be the most frequently isolated species from humans and is largely reported from South Asia. This trend perhaps reflects endemicity and the maintenance of this species in that region. Whereas *L. weilii* is largely confined to Asia, *L. santarosai* and *L. noguchi* are found to be adapted to the Americas. So far, this trend mostly holds for the isolates that have been received over the years for typing at the KIT, Amsterdam laboratory and certainly, this would have implications for understanding the transmission dynamics and evolution of the *Leptospira* or the (ubiquitous, or confined hosts) of the pathogenic species.

### Genetic affinities within the *L. interrogans* subsample

**Figure 4:**
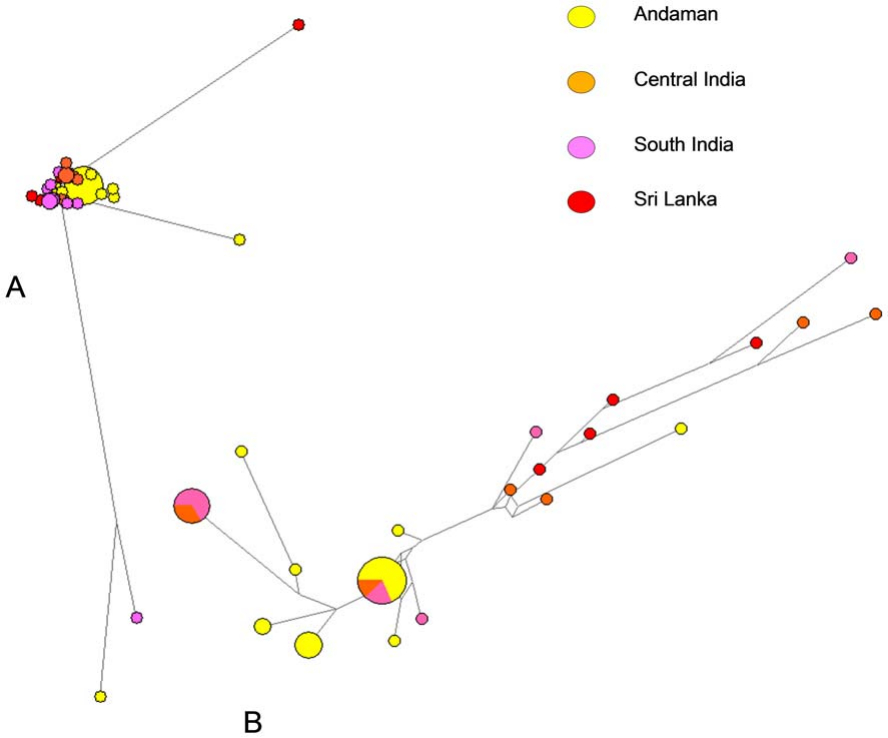
Median-joining network analysis of the MLST data obtained for the Indian (sub continent) subsample of *L. interrogans*. As evident from the trees A and B, a few sequences were highly divergent, possibly because of recombination. The enlargement of the core sequence network (B) revealed four sequences from the Andaman Islands in the central position, suggestive of a possible source of the Indian *L. interrogans* variability, followed by distinct derived clusters from South India, Central India and Andaman islands. Such a geographic structure is not apparent in the FAFLP network (Figure 1) of the Indian *L. interrogans*, confirming the reduced phylogenetic resolution of FAFLP in respect to MLST analysis. The color codes have no overlaps with those shown in Figures 1, 2 and 3. The tree/associations were deduced using Network 4.5.0.0 package.

Of the 271 rigorously sampled isolates comprising of our collection (Table S2), 134 were identified as *L. interrogans* (clade A, Figure 3). These 134 isolates were further sub classified based on MLVA to reveal that they were in fact diverse, belonging to various different serogroups, and representing different sub-ecotypes of the same species spread over the entire South Asian region (Figure 2). This was indirectly a proof that the collection did not represent convenient sampling. Further, most of these isolates (33%) were cultured in India (many from the Andaman and Nicobar islands) and were all obtained from human clinical cases as a part of routine outbreak investigations, over a period. As indicated, a majority of *L. interrogans* from our collection belonged to South Asia and were isolated from human cases of leptospirosis. Despite being geographically distinct, they formed a tight cluster. This shows their possible clonal origins and perhaps recent dispersal within the South Asian countries with less opportunity to diversify or accumulate substitutions within the candidate gene loci. Therefore, such loci appear to be conserved and stable within the clade A. In fact, it is a desirable property of the loci included in a MLST scheme that they should be more static within a particular species [27]. Given the above, it would perhaps be tempting to espouse that the *L. interrogans* ss could have been the most dominant and fittest ecotype/species to cause disease in humans in this region. A majority of these isolates were from the Andaman and Nicobar islands, where, as previously determined, leptospirosis has been traditionally endemic and has caused fatal outbreaks [28]. Not all the three schemes, MLST, FAFLP and MLVA could split the *L. interrogans* cluster based on geographic or ecological basis and thus it would be difficult to highlight its routes of spread in and out of Andaman or out of other main lands. Nevertheless, a careful exploration of the Indian isolates, based on MLST data, when analyzed by Network, revealed that a few sequences from the region were divergent, possibly because of recombination. The enlargement of the core sequence network (Figure 4) revealed four sequences from the Andaman Islands in central position, posing as a possible source of the Indian *L. interrogans* variability with distinct, derived clusters corresponding to South India, Central India and Andaman islands. Given this, it is possible to think of an early spread to and from Andaman to mainland India and to other adjoining countries, possibly through rodents that travelled in vessels and ships to Andaman from India and *vice-versa*.

### MLST as a gold standard for *Leptospira* strain typing

The six MLST loci selected and previously tested by us [8], [16] on a limited set of isolates from present collection were suitable for strain individualization. These loci could be amplified and sequenced in all the isolates (irrespective of their taxonomic status) representing pathogenic as well as saprophytic species; nonetheless, they required greater standardization for non-pathogenic variants. Among the species, these loci exhibited a high degree of sequence diversity and resolution.

Several molecular tools that have so far been described for the diagnosis of *Leptospira* are associated with drawbacks, either in the form of technical complications or the difficulties of interpretation, portability and reproducibility. Some of the methods need live organisms or a very high purity and concentration of genomic DNA. Our MLST approach overcomes all these disadvantages as the technique is simple and requires an automated DNA sequencer that is more widely available in most of the laboratories and the sequence data generated is unambiguous and explicit. The main advantage of MLST is the transfer of data that can be shared and compared between different laboratories easily through the Internet. To date a large number of organisms have been typed by MLST, which proved to be a highly discriminatory technique [29]–[31]. MLST analysis of the *Leptospira* strains confirms earlier findings [26] that the serovars and the serogroups are not clustered together but according to the species. This method is more suitable in identifying the species of leptospires as indicated by the clustering patterns up to genome species level. Due to the greater sequence diversity observed in all the six genes except *rrs*2, the dendrogram generated could differentiate effectively the *L. interrogans* ss, *L. kirschneri*, *L. noguchi*, *L. weilii*, *L. santarosai* and *L. borgpetersenii*. Thus, our MLST technique and its analysis by Bayesian MCMC were capable of individualizing *Leptospira* up to species level with flexibility to type isolates with many different taxonomic identities as compared to another MLST scheme [17] which has been limited to outbreak investigation(s) over small epidemiological territories and could not type isolates beyond *L. interrogans* ss. Having said this, we should also consider the obvious limitation of MLST: its failure to resolve the horizontal variome [32], but this really depends on the extent and impact of HGT in different bacterial species. With this issue in mind, we already included targets other than the housekeeping genes, namely, the envelope proteins, LipL32 and LipL41 in our MLT scheme [8], [16]; this may allow sampling of variation beyond the core genome and which might be relevant in epidemiologic/taxonomic resolution of the strains.

### The future of *Leptospira* genotyping

With our extensive evaluation of the MLST technique and its comparison with FAFLP, we believe that the issues related to strain diversity as well as the taxonomic organization and accuracy of the reference collection(s) were set to rest in a best possible way. This will help understand population genetic structure of this pathogen with diverse host range and under different ecological conditions and will provide a scope for genotype-phenotype correlation to be established. Analyses based on the allelic profiles generated by MLST could be successfully used to gain insights into the evolution and phylogeographic affinities of leptospires as it has been done for many other organisms. Given the associations and affinities within our collection, it will be possible in the foreseeable future to develop a sophisticated database of the genomic profiles based on all the three typing techniques. Finally, our rigorous categorization of the ecotypes and genotypes herein may be seen as the first, needed step under the mandate of the post genomic profiling of *Leptospira* from different hosts [33]. This will help the leptospirosis community in planning for future whole genome sequencing [34] of Leptospirae or establishing their metagenome. Such approaches will be able to generate extremely valuable information in the form of diagnostic markers, vaccine candidates, and strain specific co-ordinates relevant in re-constructing the evolutionary history of the organisms emerging or reemerging in a particular epidemiological catchment area. This reality ultimately holds promise for strengthening the cause of ‘functional molecular infection epidemiology (FMIE)’ of *Leptospira*. FMIE is an emerging area of medical microbiology that entails correlation of genetic variation in a pathogen, with a unique function in corresponding host(s) related to disease severity, disease progression, or host susceptibility. This kind of functional epidemiology is likely to explain not only the genome level, descriptive, host-pathogen associations, but also the global juxtaposition of pathogen and host variations with a prospective impact on our understanding of pathogen/infection biology.

In conclusion, our integrated genotyping approach provides evidence that *Leptospira* represent a globally distributed zoonotic agent and their gene pool being diverse and somewhat geographically compartmentalized. In addition, *L. interrogans* appears to be the single, most prevalent *Leptospira* species, which inflicts rodents, livestock and humans in different continents but predominantly in the South Asian countries. While we found FAFLP to be an important method for outbreak investigation and for clustering of isolates based on their geographical descent rather than by genome species types, it was not able to convey much taxonomical utility sufficient to replace tedious serotyping procedures, currently in practice, worldwide. By contrast, MLST was observed to be highly robust and efficient in identifying ancestral relationships and segregating the outbreak associated strains according to their genome species status. We believe that this large-scale evaluation of different genotyping methods sets stage for the implementation of MLST as a highly sought-after replacement of serotyping, for accurate identification and classification of *Leptospira*.

## Supporting Information

Table S1 Characteristics, distribution and phylogenetic affiliation of *Leptospira* isolates. Species short names *L. int*, *L. borg*, *L. kirsch*, *L. sant*, and *L. nog* refer to *L. interrogans ss*, *L. borgpetersenii*, *L. kirschneri*, *L. santarosai* and *L. noguchi*, respectively. Short names of serogroups, Gripp., Ict., Aust., and Cani. refer to Grippotyphosa, Icterohaemorrhagiae, Australis, and Canicola respectively.
(0.15 MB DOC)

Table S2 Full details and multilocus sequences of the *Leptospira* isolates and strains used in this study.
(0.39 MB XLS)
