# Supplementary material for: Genetic Affinities within a Large Global Collection of Pathogenic Leptospira: Implications for Strain Identification and Molecular Epidemiology
Source: PLoS One. 2010 Aug 27;5(8):e12637. doi: 10.1371/journal.pone.0012637 (PMC2929200; doi:10.1371/journal.pone.0012637)
Supplement: Table S1 — Characteristics, distribution and phylogenetic affiliation of Leptospira isolates. Species short names L. int, L. borg, L. kirsch, L. sant, and L. nog refer to L. interrogans ss, L. borgpetersenii, L. kirschneri, L. santarosai and L. noguchi, respectively. Short names of serogroups, Gripp., Ict., Aust., and Cani. refer to Grippotyphosa, Icterohaemorrhagiae, Australis, and Canicola respectively. (0.15 MB DOC) [file pone.0012637.s001.doc]

| **S. No** | **Region** | **Country** | **No. of Isolates** | | **% of total collection** | **Predominent species** | **Major serogroups** | **Major Source/Host** | **Major MLST clade(s)** |
| --- | --- | --- | --- | --- | --- | --- | --- | --- | --- |
| 1 | South Asia |  |  | 94 | 34.7 | *L. int., L. borg.* | Gripp., Ict., Aust., Cani. | Human (74) | A |
|  |  | India | 54 |  |  |  |  |  |  |
|  |  | Indonesia | 14 |  |  |  |  |  |  |
|  |  | Sri Lanka | 6 |  |  |  |  |  |  |
|  |  | Malaysia | 15 |  |  |  |  |  |  |
|  |  | Philippines | 3 |  |  |  |  |  |  |
|  |  | Vietnam | 2 |  |  |  |  |  |  |
| 2 | Rest of Asia |  |  | 24 | 9 | *L. weilii* | Javanica | Human (9) | G2 |
|  |  | China | 14 |  |  |  |  |  |  |
|  |  | Kazakhstan | 1 |  |  |  |  |  |  |
|  |  | Korea | 2 |  |  |  |  |  |  |
|  |  | Russia | 5 |  |  |  |  |  |  |
|  |  | Israel | 1 |  |  |  |  |  |  |
|  |  | Kuwait | 1 |  |  |  |  |  |  |
| 3 | Europe |  |  | 26 | 9.5 | *L. borg, L. int.* | Sejroe | Mainly rodents/animals (12) | A, G |
|  |  | Bulgaria | 3 |  |  |  |  |  |  |
|  |  | Czech Republic | 1 |  |  |  |  |  |  |
|  |  | Slovakia | 1 |  |  |  |  |  |  |
|  |  | Denmark | 4 |  |  |  |  |  |  |
|  |  | Germany | 1 |  |  |  |  |  |  |
|  |  | Ireland | 7 |  |  |  |  |  |  |
|  |  | United Kingdom | 3 |  |  |  |  |  |  |
|  |  | Italy | 2 |  |  |  |  |  |  |
|  |  | Portugal | 3 |  |  |  |  |  |  |
|  |  | Romania | 1 |  |  |  |  |  |  |
| 4 | Africa |  |  | 34 | 12.5 | *L. borg., L. kirsch., L. int* | Ballum | Mainly rodents (25) | G, K |
|  |  | Ghana | 1 |  |  |  |  |  |  |
|  |  | Kenya | 4 |  |  |  |  |  |  |
|  |  | Tanzania | 23 |  |  |  |  |  |  |
|  |  | Tunisia | 1 |  |  |  |  |  |  |
|  |  | Congo (Zaire) | 3 |  |  |  |  |  |  |
|  |  | Zimbabwe | 2 |  |  |  |  |  |  |
| 5 | North/Central America |  |  | 35 | 13 | *L. sant., L. int., L. nog.* | Tarassovi | Opossum (10), Human (10) | A, D, J |
|  |  | Costa Rica | 10 |  |  |  |  |  |  |
|  |  | USA | 9 |  |  |  |  |  |  |
|  |  | Nicaragua | 3 |  |  |  |  |  |  |
|  |  | Panama | 11 |  |  |  |  |  |  |
|  |  | Jamaica | 2 |  |  |  |  |  |  |
| 6 | South America and Caribbean |  |  | 35 | 13 | *L. sant, L. int.* | diverse | Opossum (11), Livestock (10) | A, J |
|  |  | Trinidad and Tobago | 3 |  |  |  |  |  |  |
|  |  | Argentina | 1 |  |  |  |  |  |  |
|  |  | Brazil | 19 |  |  |  |  |  |  |
|  |  | Puerto Rico | 2 |  |  |  |  |  |  |
|  |  | Peru | 10 |  |  |  |  |  |  |
| 7 | Australia |  |  | 7 | 2.5 | *L. int.* | Pyrogenes | Human (3) | A |
|  |  | Australia | 6 |  |  |  |  |  |  |
|  |  | New Guinea | 1 |  |  |  |  |  |  |
|  |  |  |  |  |  |  |  |  |  |
